# A Scoping Review on the Polymerization of Resin-Matrix Cements Used in Restorative Dentistry

**DOI:** 10.3390/ma16041560

**Published:** 2023-02-13

**Authors:** Rita Fidalgo-Pereira, Orlanda Torres, Óscar Carvalho, Filipe S. Silva, Susana O. Catarino, Mutlu Özcan, Júlio C. M. Souza

**Affiliations:** 1 University Institute of Health Sciences (IUCS), CESPU, 4585-116 Gandra, Portugal; 2 Center for Interdisciplinary Research in Health (CIIS), Faculty of Dental Medicine (FMD), Universidade Católica Portuguesa (UCP), 3504-505 Viseu, Portugal; 3 Center for MicroElectroMechanical Systems (CMEMS-UMINHO), University of Minho, Campus Azurém, 4800-058 Guimarães, Portugal; 4 LABBELS Associate Laboratory, University of Minho, 4805-017 Guimarães, Portugal; 5 Division of Dental Biomaterials, Center of Dental Medicine, Clinic of Reconstructive Dentistry, University of Zurich, 8032 Zurich, Switzerland

**Keywords:** degree of conversion, resin cement, light transmittance, polymerization, light curing

## Abstract

In dentistry, clinicians mainly use dual-cured or light-cured resin-matrix cements to achieve a proper polymerization of the organic matrix leading to enhanced physical properties of the cement. However, several parameters can affect the polymerization of resin-matrix cements. The main aim of the present study was to perform a scoping review on the degree of conversion (DC) of the organic matrix, the polymerization, and the light transmittance of different resin-matrix cements used in dentistry. A search was performed on PubMed using a combination of the following key terms: degree of conversion, resin cements, light transmittance, polymerization, light curing, and thickness. Articles in the English language published up to November 2022 were selected. The selected studies’ results demonstrated that restorative structures with a thickness higher than 1.5 mm decrease the light irradiance towards the resin-matrix cement. A decrease in light transmission provides a low energy absorption through the resin cement leading to a low DC percentage. On the other hand, the highest DC percentages, ranging between 55 and 75%, have been reported for dual-cured resin-matrix cements, although the polymerization mode and exposure time also influence the DC of monomers. Thus, the polymerization of resin-matrix cements can be optimized taking into account different parameters of light-curing, such as adequate light distance, irradiance, exposure time, equipment, and wavelength. Then, optimum physical properties are achieved that provide a long-term clinical performance of the cemented restorative materials.

## 1. Introduction

A reliable and lasting indirect restoration is the main aim of oral rehabilitation, although that depends on the performance of resin-matrix cements [1]. The polymerization of resin-matrix cements under ceramic or resin-matrix composite restorations plays a key role in the mechanical properties of the restorative interface [2,3]. An inadequate polymerization promotes a faster degradation of resin-matrix cement and increases the risk of fracture at the restorative interface, leading to debonding [4]. Transmitted light irradiance has a strong effect on the degree of conversion (DC) of monomers within the organic matrix of resin-matrix cements [5,6]. In fact, the DC of resin-matrix cements directly affects their physical properties, and it has been used as a parameter for predicting the restorations’ clinical performance [7]. The effects of light transmittance on the polymerization of resin-matrix cements should be clarified concerning their mechanical performance and biocompatibility.

The most common commercially available resin-matrix cements often used by clinicians are dual-cured and self-adhesive cements [7,8]. In particular, dual-cured resin-matrix cements have become the most used materials due to light-curing and chemical polymerization under light-curing procedures [9,10]. Regarding polymerization, there are three types of resin-matrix cements: light curing, self-curing, and dual curing [11,12]. Resin-matrix cements are composed of an organic matrix which agglomerates dispersed inorganic fillers. The organic matrix often involves bisphenol A-glycidyl dimethacrylate (Bis-GMA), urethane dimethacrylate (UDMA), and triethylene glycol dimethacrylate (TEGDMA). Inorganic fillers are composed of combinations of barium fluoroaluminoborosilicate, strontium calcium aluminosilicate glass, quartz, amorphous silica, ytterbium fluoride, zirconia, and other glass fillers [13,14]. The content of inorganic fillers of commercially available resin-matrix cements varies in the range from 60 up to 75 wt% [13,15].

Tooth-supported restorations can involve different restorative materials, resin-matrix cements, and tooth substrate conditions [7]. The most frequent materials applied on indirect restorations are zirconia, feldspar-based ceramics, lithium disilicate-reinforced glass ceramics, zirconium-lithium silicate glass-ceramics, resin-matrix composites, and polymer-infiltrated ceramic networks (PICN) [16,17]. Indirect restorative materials have different mechanical properties that influence the clinical performance of the restoration. The elastic modulus of zirconia can reach mean values of 240 GPa [18,19] and its flexural strength ranges between 900 and 1200 MPa [20]. Translucent zirconia has an elastic modulus of around 200–210 GPa [21,22] and a flexural strength between 500 and 600 MPa [21,23]. High-translucency zirconia shows an intermediate translucency between the traditional opaque zirconia and lithium disilicate-reinforced glass ceramics [24]. Lithium disilicate-reinforced glass ceramics have an elastic modulus around 65 GPa and a flexural strength of around 380 MPa. The lowest values of elastic modulus and flexural strength have been reported for resin-matrix composites (16 GPa and 240 MPa, respectively) and PICN (21 GPa and 200 MPa, respectively) [20,25,26,27]. The adhesion of resin-matrix cements to indirect restorations depends on the surface condition of the substrate [28,29]. Several methods of surface modification have been assessed in the literature such as acidic etching (e.g., 10% HF), grit-blasting with alumina or silica micro-scale particles, and silane conditioning [30,31,32,33]. Previous studies have reported an increase in the shear bond strength of resin-matrix cements to indirect restorations related to an increase in the roughness of the substrate after surface treatment. Additionally, a chemical functionalization of the substrate enhances the adhesion between the resin-matrix cement and the ceramic substrate. Certain compounds such as silane can establish a chemical bonding between the carbon-based structure of the resin-matrix cement and the inorganic restorative materials such as zirconia or glass-ceramics. Novel studies have shown an increased adhesion of resin-matrix composites to ceramic and polymeric surfaces due to previous surface conditioning with compounds containing graphene oxide or carbon nanotubes [28,29].

Polymerization of resin-matrix cements can be affected by the type, thickness, refraction index, and shade of indirect restorations, which influence the materials’ translucency [5,34,35]. The light-curing mode and light intensity of light-curing units (LCU) also influence the polymerization process, since light irradiance decreases as the restoration thickness and shade increase [4,36]. A low DC of the organic matrix of the resin-matrix cement is also dependent on their properties [19]. The DC percentage of resin-matrix cements is dependent on the restorative material thickness and translucency, which influence the amount of light transmission towards the resin-matrix cement under an indirect restoration. The light irradiance over the light curing process decreases when the ceramic material thickness is higher than 1 mm [8]. A previous study analyzed the DC of resin-matrix cements through different high-translucency zirconia shades with 0.9 mm thickness. Higher values of DC were measured for translucent zirconia when compared to opaque ones [37]. The thickness of lithium disilicate-reinforced glass ceramics (between 0.6 and 1.5 mm) did not influence the DC values of dual-cured or light-cured cements [38]. However, different results were found when the thickness of the lithium disilicate-reinforced glass ceramic was higher than 2 mm with a significant decrease in DC percentage for resin-matrix cements [39].

Thus, the adequate polymerization of resin-matrix cements is a key factor to avoid clinical failures of indirect restorations. The main aim of this study was to perform a scoping review on the degree of conversion of the organic matrix, polymerization, and light transmittance through different resin-matrix cements used in dentistry. It was hypothesized that the polymerization mode, type of resin cement, restorative material, and light exposure time have a significant effect on the degree of monomer conversion.

## 2. Method

A bibliographic search was performed on PubMed (via National Library of Medicine) considering that includes most journals in the field of biomaterials and dentistry. The bibliographic search and the selection of studies were in accordance with the method used in previous integrative review studies [15,40,41,42,43]. The following search terms were applied: “degree of conversion” AND “resin cement” AND “light transmittance” OR “light absorption” AND “light curing mode” OR “polymerization mode“ AND “restoration thickness” OR “restoration type”. The inclusion criteria were articles published in the English language up to November 2022, focusing on the degree of conversion of the organic matrix, polymerization, and light transmittance through different resin-matrix cements used in dentistry. The eligibility inclusion criteria also included in vitro studies, randomized controlled trials, animal assays, and prospective cohort studies. The exclusion criteria were the following: papers without an abstract; pilot studies; case reports with a short follow-up period; and studies with a lack of details on the polymerization of resin-matrix cements. Additionally, a hand-held search was also performed on the reference lists of all primary sources and eligible studies. Studies were not restricted based on publication date during the search process.

The articles retrieved from the search process were evaluated in three steps. All articles were compiled for each combination of key terms and therefore the duplicates were removed using Mendeley citation manager (Elsevier BV). Studies were primarily scanned for relevance by title, and the abstracts of those that were not excluded at this stage were assessed. The second step comprised the evaluation of the abstracts and non-excluded articles according to the eligibility criteria on the abstract evaluation. Two of the authors (JCMS and R.F-.P) independently evaluated the titles and abstracts of potentially relevant articles. A preliminary evaluation of the abstracts was carried out to establish whether the articles met the main aim of the study. Selected articles were individually read and evaluated concerning the purpose of this study. At last, the eligible articles received a study nomenclature label, combining first author names and year of publication. Two reviewers independently organized the data, for example, by author name, journal, publication year, purpose, resin-matrix cement data, restoration types, and degree of conversion.

## 3. Results and Discussion

In this study, the main parameters related to the adequate polymerization of resin-matrix cements are discussed, such as the degree of conversion of the organic matrix, light curing procedures, light irradiance, and light transmittance. The polymerization of resin-matrix cements is affected by the content, type, and size of inorganic fillers. The photoinitiator system also plays a key role in the polymerization chain reaction in the organic matrix. On restorations, opaque restorative materials decrease the light transmittance towards the resin-matrix cement and therefore negatively affect the degree of conversion of the organic matrix. The highest degree of conversion in dual-cured cements when compared to light-cured cements was recorded with soft-start polymerization. Thus, the findings of the selected studies validate the hypothesis of the present review.

**Figure 1:**
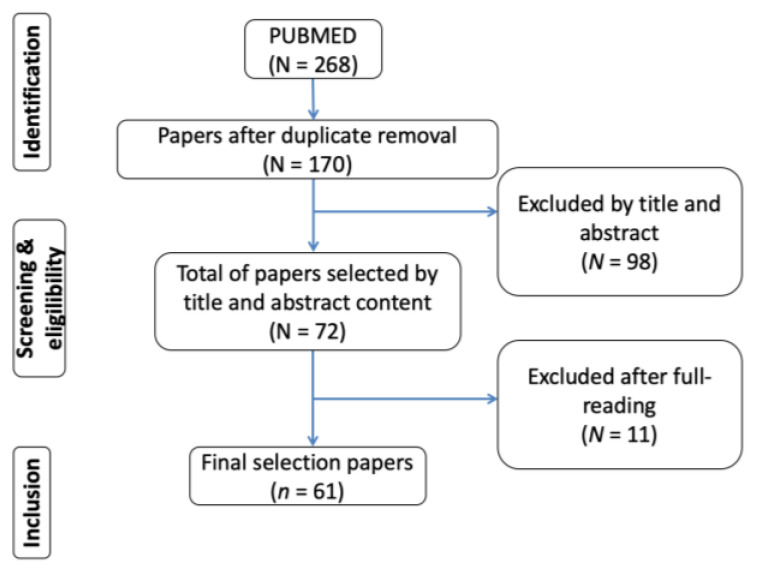
Schematics on the search and selection of articles.

The initial search in the available database yielded a total of 268 articles, of which 98 duplicate articles were eliminated. Of the remaining 170 articles, the titles and abstracts were read seeking concordance with the inclusion criteria of the present study and then 11 studies were discarded because of the lack of data. The evaluation of titles and abstracts resulted in the selection of 61 potential review articles of which six articles were excluded. The results of the selection of articles are shown in Figure 1.

### 3.1. The Chemical Composition of Resin-Matrix Cements 

The chemical composition of resin-matrix composite cements is quite similar to that recorded for resin-matrix composites, since they are composed of an organic matrix reinforced with silanated inorganic fillers [44,45]. Most resin-matrix cements contain high molecular weight monomers (Bis-GMA, UDMA, TEGDMA, and hydroxyl ethyl methacrylate (HEMA), as described in Table 1 [46]. Bis-GMA has a high molar mass (512 g mol^−1^) and a high viscosity (600–1000 Pa.s^−1^), which limits the addition of inorganic particles to resin-based materials [41,47,48]. Additionally, UDMA is commonly used as a base monomer in self-adhesive resin-matrix cements in combination with TEGDMA [49]. Current self-adhesive resin cements are composed of conventional dimethacrylate monomers such as Bis-GMA, UDMA, HEMA, urethane oligomers of Bis-GMA, glycerol dimethacrylate, TEGDMA, or trimethyloylpropane trimethacylate (Table 1). Self-adhesive resin cements are also composed of acid-functionalized monomers, which are used to achieve demineralization and bonding to the tooth surface, e.g., 4-methacryloyloxyethyl trimellitic anhydride (4-META) or phosphoric acid groups, such as 10-methacryloyloxydo-decyl dihydrogen phosphate (MDP), 2-methacryloxyethyl phenyl hydrogen phosphate (Phenyl-P), bis(2-methacryloxyethyl) acid phosphate (BMP), and dipentaerythritol pentaacrylate monophosphate (Penta-P) [13,50]. However, the concentration of the acidic monomers in self-adhesive resin cements must be balanced regarding hydrophilicity and pH to establish an effective bonding to both the dentin and enamel surfaces [45,46,51]. Camphorquinone (CQ) is also included as a photoinitiator and is associated with a coinitiator such as tertiary amine (TA) (Table 1). The CQ/TA complex is stimulated by visible light from light-curing to provide free radicals responsible for initiating the polymerization chain reaction [48,52,53]. Nevertheless, the low color stability of amines remains an issue, and therefore other photoinitiator systems can be found in light-cured resin cements, such as germanium-based photoinitiators Ivocerin^TM^ (bis(4-methoxybenzoyl)diethylgermanium) and Lucirin^TM^ TPO (2,4,6-trimethylbenzoyldiphenyl phosphine oxide) [52,54].

Regarding the inorganic composition, commercially available resin-matrix cements contain an inorganic content ranging from 57 up to 78 wt% [13,14,19,55,56,57,58,59,60]. As shown in Table 1, colloidal silica, ytterbium, or barium glass are the most frequent inorganic particles used as fillers [19,61]. However, other combinations of fillers have also been used, such as fluoroaluminoborosilicate glass, strontium calcium aluminosilicate glass, quartz, or other glass fillers [13]. The inorganic filler content is responsible for enhanced mechanical properties, although resin-matrix cements usually have a lower filler content when compared with resin-matrix composites [58,61]. Resin-matrix cements reveal a high variability in shape, size, and content of inorganic filler particles [56]. Commercially available resin-matrix cements can include different particles sizes and types of spherical- and irregular-shaped particle inorganic fillers [57]. Inorganic particles of three self-adhesive resin cements (Calibra^TM^, Denstply; MaxCem Elite^TM^, Kerr; and RelyX U200^TM^, 3M ESPE) have mean particle sizes of 5.95 μm, 9.23 μm, and 8.1 μm, respectively. The highest flexural strength values of around 96 MPa were recorded for a resin-matrix cement with a high content of micro-scale inorganic fillers [59]. Other resin-matrix cements have inorganic fillers with sizes ranging from 0.5 up to 3 μm [14]. The incorporation of submicron- and nano-scale fillers has also shown improvements in the physical properties of resin cements [62]. A previous study reported the influence of nanoparticle content on the properties of hybrid resin luting agents, and concluded that the incorporation of nanoparticles improves the properties of resin-matrix cements [63]. The film thickness of resin-matrix cements is also important as it improves the restoration fitting and mechanical performance, while decreasing marginal leakage and loss of marginal integrity. The resin-matrix layer thickness can be affected by multiple factors, such as viscosity, inorganic filler content, organic composition, and DC. The thickness limit of a resin-matrix cement is clinically acceptable below 50 μm following the ISO standard [64].

### 3.2. Polymerization Pathways 

The polymerization reaction of chemically cured (self-cured) resin cements occurs solely by activation of tertiary amines and benzoyl peroxide [65]. Chemically cured (self-cured) cements are used for thick restorations, luting posts, and crowns that block light transmission, such as metallic materials or highly opaque ceramics. Regarding the self-cure reaction, the initiator, namely benzoyl peroxide, chemically bonds to a co-initiator (tertiary amine), providing free radicals within the polymeric chain [11]. Resin-matrix cements include benzoyl peroxide as a chemical initiator in one paste, while a second paste contains the tertiary amine [66]. To ensure the success of the polymerization process, an increased concentration of redox initiators is required to achieve the high DC of monomers. However, chemically cured resin-matrix cements have the drawbacks of a limited working time and a prolonged setting time [67]. The chemical reaction involves a prolonged working time and could compromise the luting procedure [10]. 

Light-cured cements are activated in the presence of a visible light source using light-curing units (LCU) [68]. The CQ/TA complex is stimulated by visible light at wavelengths ranging from 420 up to 490 nm [69], although the light absorption peak is recorded at 470 nm (Table 1) [52,53]. Regarding the light-curing reaction, blue visible light (at 470 nm) transmitted by a LCU is absorbed by the CQ/TA complex, involving three fundamental steps: (1) polymeric chain growth without restrictions, in which the monomer concentration and free radicals determine the kinetic reactions; (2) fast viscosity increase with restricted mobility of free radicals and an irreversible increase in the elastic modulus of the resin matrix (gel point); and (3) vitrification and end of the reaction with a high viscosity level [41,47,48,70]. The increase in viscosity limits the movements of the remaining monomers responsible for additional polymerization [71,72,73]. Dual-cured resin-matrix cements combine light and chemical activation to ensure adequate polymerization under low light irradiance [10]. Dual-cured resin-matrix cements also include benzoyl peroxide as a chemical initiator in one paste [66], while a second paste contains a photoinitiator and TA. A chemical reaction occurs between the benzoyl peroxide and the tertiary amine, while the photoinitiator system (CQ/TA) is also stimulated by visible light [74]. Regarding polymerization, self-adhesive resin cements contain an acidic monomer that may compromise the sufficient polymerization of the resin-matrix cements, diminishing the physical properties [75]. 

Regarding the photoinitiator type, Lucirin^TM^ TPO, which has a lower wavelength of light absorption ranging from 380 up to 425 nm, has shown higher DC values, higher color stability, and improved resistance to hydrolytic degradation when compared to CQ [16,76]. However, several studies have reported processes to improve the performance of CQ as a photoinitiator. A previous study reported the use of idonium salt as a component of the initiator system to increase the DC of the resin-matrix cement in regions with low light exposure [77]. The combination of CQ and idonium salt increased the number of free radicals formed per molecule of CQ and allowed a higher DC percentage [78,79]. The development of LCU with multiple-emssion LEDs allows a correct match with new photoinitiators system sensitive to the violet wavelength range, which are often combined with CQ. The spectral emission of single- or multiple-emission LCU must be selected to stimulate the initiator system [80]. Studies have shown that dual-cured resin cements have higher DC percentages than those of light-cured cements [81,82]. Comparing two dual-cured resin cements, RelyX ARC^TM^ (3M ESPE) showed a DC percentage of 72.8% (3.7 ^a^) and Variolink II^TM^ (Ivoclar Vivadent) showed a DC percentage of 65.7% (2.9 ^a^). Assessing only the paste containing the photoinitiator, RelyX ARC^TM^ showed a DC percentage of 57.5% (4.4 ^c^) and Variolink II showed a DC percentage of 58.3% (2.1 ^c^). The DC percentage of a light-cured resin-matrix cement (Variolink^TM^) was recorded at 48.6% (4.1 ^a^). The polymerization was performed through a 1 mm-thick felspar-based ceramic with visible light at 800 mW/cm^2^ from a QTH LCU for 40 s. The DC percentage was measured by Fourier transform infrared spectroscopy (FTIR) [74] (Table 1).

**Table 1:** Relevant data retrieved from previous studies on resin-matrix cements and their degree of conversion.

Author (Year)	Purpose	Resin-Matrix Cement	Light-Curing Procedures	Degree of Conversion (%)	
Turp et al. (2015) [36]	Evaluation of the effect of the thickness of zirconia on the curing efficiency of resin cements.	10- MDP, DMA, Bis-MPEPP (25%); silanized barium glass (75%) (Panavia F 2.0, Kuraray, Japan)	LED (Elipar S10, 3M, ESPE, Saint Paul, MN, USA) for 20 s, 430–480 nm, 1200 W/cm^2^	(G0) 69.95; 62.67; 53.15 (G1) 65.26; 58; 49 (G2) 62.8; 54.15; 43.64 (G3) 52.1; 49.33; 39.41	
Lopes et al. (2015) [83]	Evaluation of the degree of conversion (DC), Vickers microhardness (VH), and elastic modulus (E) of resin cements	Bis-GMA, Bis-EMA, TEGDMA (35%); barium alumo-silicate glass, silicon dioxide (66%); (Allcem, FGM, Brazil) Bis-GMA, urethane dimethacrylate, and triethylene glycol dimethacrylate. (56.4%); barium glass, ytterbium trifluoride, ba-Al-fluorosilicate glass, and spheroid mixed oxide (43.6%); (Variolink II, Ivoclar Vicadent, Liechsteintein) Methacrylate monomers (28%); silanated fillers, alkaline fillers. (72%); (RelyX U200, 3M ESPE, USA) Bis-GMA, UDMA, Bis-EMA, HEMA (60.3%); barium glass, ytterbium trifluoride, spheroid mixed oxide (39.7%); (Multilink, Ivoclar Vicadent, Liechsteintein)	Conventional halogen light-curing (Optilux) for 120 s; 501–650 mW/cm^2^	(G0) 74.4; (G1) 71.1 (G0) 60.7; (G1) 67.9 (G0) 70; (G1) 76.2 (G0) 44; (G1) 43.7	
Sulaiman et al. (2015) [84]	Evaluation of the influence of material thickness on light irradiance, radiant exposure, and the DC of two dual-polymerizing resin cements light-polymerized through different brands of monolithic zirconia	Methacrylate monomers (57%); Silanated fillers (43%); (RelyX Ultimate, 3M ESPE, USA) Bis-GMA, urethane dimethacrylate, and triethylene glycol dimethacrylate. (56.4%); Barium glass, ytterbium trifluoride, Ba-Al-fluorosilicate glass, and spheroid mixed oxide (43.6%); (Variolink II, Ivoclar Vicadent, Liechsteintein)	(G1) LED (Elipar S10) for 20 s; 1200 mw/cm^2^; 430–480 nm. (G2) LED (Elipar S10) for 40 s; 1200 mw/cm^2^; 430–480 nm.	(G1) 63.1 (G2) 66	
Gültekin et al. (2015) [34]	Evaluation of the polymerization efficiency of a dual-cured resin cement cured with two different light curing units under zirconia structures with differing thicknesses	10- MDP, DMA, Bis-MPEPP (25%); silanized barium glass (75%); (Panavia F 2.0, Kuraray, Japan)	LED (Elipar S10, 3M ESPE, Seefeld, Germany) for 20 s (5 s rmp, 15 s full cure); 430–480 nm; 1200 mW/cm^2^ QTH (Hilux 200, Benlioglu, Istanbul, Turkey) for 40 s (time in continuous mode); 410–500 nm; 600 mW/cm^2^	QTH: (Z) 66.7; 60; 48.7 (Z1) 58.8; 54.3; 44.1 (Z2) 52.7; 48.2; 41.3 (Z3) 49.3; 46.2; 37.8	LED: (Z) 69.9; 62; 53 (Z1) 65.2; 58; 49 (Z2) 62.8; 54.1; 43.6 (Z3) 52.1; 49.3; 39.4
Shim et al. (2017) [50]	Evaluation of the polymerization mode of self-adhesive, dual-cured resin cements light-cured through overlying materials with different degrees of translucency by measuring the DC.	UDMA; fluoro alumino silicate glass; (G-CEM Link ACE, GC Corp, USA) Bis-GMA; Fluoro aluminio silicate glass, fumed silica, barium glass, ytterbium fluoride (Maxcem Elite, Kerr Dental, USA) Bis-GMA; dental glass; (BisCem, Bisco, USA)	LED (Dr’s Light; Good Doctors Co., Incheon, Korea) for 40 s; 718 mW/cm^2^	50–75	
Caprak et al. (2018) [85]	Evaluation of the influence of the translucency parameters (TPs) of current monolithic CAD/CAM blocks on the microhardness of light-cured or dual-cured resin cements.	Bis-GMA, UDMA, TEGDMA; glass fillers; (Bisco Duo-Link, Bisco, USA)	LED (HS-LED1500; Henry Schein, Ontario, Canada) for 40 s; 1500 mW; 450–470 nm	Dual-cured: (G0) 59; (G1) 53 (G0) 57; (G1) 52 (G0) 56; (G1) 49 (G0) 56; (G1) 48	Light-cured: (G0) 56; (G1) 48 (G0) 54; (G1) 46 (G0) 53; (G1) 45 (G0) 53; (G1) 43

The light-curing process of dual- and light-cured resin-matrix cements remains crucial to the overall DC efficacy. As previously noted, the DC directly influences the physical properties of resin-matrix cements [86,87]. The DC and shrinkage behavior are important factors in the clinical selection of resin-matrix cements [48,88]. The DC percentage of a resin-matrix cement through an indirect restoration is typically measured to be between 55 and 75%, as seen in Table 1 [2,65]. An insufficient DC percentage of monomers can increase the solubility, causing microleakage at margin restorations [89]. Thus, a high DC and an efficient polymerization depend on several factors such as (i) light irradiance, (ii) exposure time [90], (iii) visible light wavelength, (iv) organic matrix type, size, and content of inorganic fillers, (v) distance between the LCU and resin-matrix cement [91], and (vi) the refraction index of the organic and inorganic components [40,92]. The amount of light received by the resin-matrix cement is dependent on the LCU. Failures during the light curing process may lead to indirect restoration debonding, toxicity, increased marginal wear, and bacterial accumulation [80]. 

**Figure 2:**
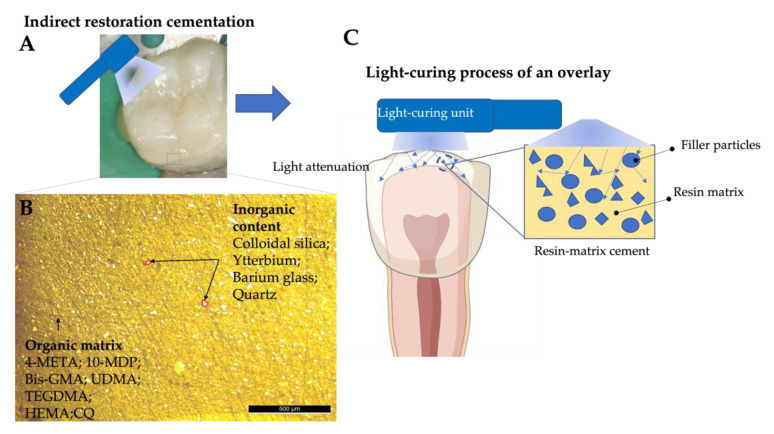
**A**—Overlay cementation; **B**—Optical microscope image, 50× magnification, showing the organic matrix and the inorganic content; **C**—Schematics of light scattering.

Light attenuation and scattering phenomena are dependent on the filler volume fraction, particle size, fillers shape, and the refractive indices of the materials, as illustrated in Figure 2 [7,93]. A previous study [10] developed an experimental protocol in which thiourethane was added to dual-cured cements, and the decreases in the contents of two photoinitiators, namely Bis-acylphosphine oxide (BAPO) and p-Tolyldiethanolamine (DHEPT), were measured. The resin cements showed an improved DC, a reduction in polymerization stress, and had a long working time. Additionally, the delay in the light-activation of the dual-cured cements did not affect the DC, the flexural strength, or the polymerization stress, showing a limited effect on their elastic modulus [10]. In another previous study, three resin-matrix cements were polymerized under different glass ceramics and zirconia with an LED at 650 mW/cm^2^ for 60 s. The results revealed the following DC percentages: 64.5% for Variolink^TM^; 69.5% for RelyX U200^TM^; and 59.1% for Multilink^TM^. The lowest DC percentage was related to the chemical composition of the resin-matrix cement, since the amount of chemical initiators was limited to allow a long working time. DC measurements were performed by attenuated total reflectance/Fourier transform infrared spectroscopy (ATR/FTIR) [83]. 

### 3.3. The Influence of Indirect Restorative Materials

Ceramic materials provide a natural appearance, biocompatibility, chemical stability, high compressive resistance, and thermal expansion similar to those of tooth structures [94]. Furthermore, high bond strength values have been reported on the assembly established by indirect ceramic restorations, resin-matrix cement, and tooth hard tissues [95]. Among ceramics, lithium disilicate-reinforced glass ceramics are the most frequently used due to its excellent esthetic and improved mechanical properties [94]. A previous study compared the light transmittance through different thickness (0.5, 1.0, 2.0, and 4.0 mm) of two glass-ceramic materials: a high translucency (HT) leucite-reinforced glass ceramic (IPS Empress HT^TM^, Ivoclar Vivadent) and a low translucency (LT) lithium disilicate glass ceramic (IPS e.max LT CAD^TM^, Ivoclar Vivadent) [4]. The light transmittance values of the HT leucite-reinforced glass ceramic were higher when compared to the lithium disilicate-reinfored glass ceramic. The DC values of resin-matrix cement were also higher for the HT leucite-reinforced glass ceramic when compared with the LT lithium disilicate-reinforced glass ceramic [4]. Those findings were corroborated by other studies [3,35,96,97,98]. Thus, the chemical composition of lithium disilicate-reinforced glass ceramics (70 wt% lithium disilicate crystal), consists of small, randomly oriented, interlocking, plate-like crystals. Light transmittance is impaired by the plate-like crystals. Considering that the ceramic type can cause a significant difference in light transmittance, such difference might have to be considered when choosing the ceramic type for deep cavities, where light transmittance is a major issue.

**Figure 3:**
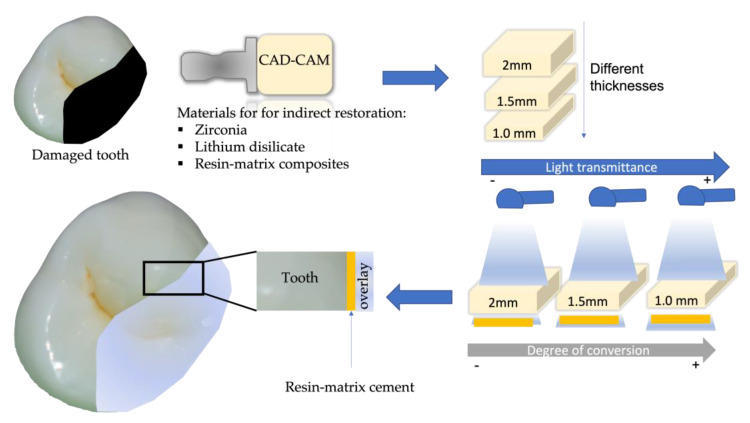
Schematics on the influence of different indirect restorative materials and material thicknesses on light transmittance and the degree of conversion of resin-matrix cement.

The shade and thickness of the indirect restoration also affect the translucency parameters and the light transmission towards the resin-matrix cement [3,5]. The chemical composition and opacity of the restorative material attenuate light intensity and reduce the number of photons reaching the resin-matrix cement, possibly compromising the indirect restoration prognosis [72,84,85,99,100,101]. In clinical applications, the light-curing parameters should be controlled regarding the thickness and microstructure of opaque prosthetic structures. For instance, the light-curing intensity and time should be increased for thick and opaque zirconia, as shown in Figure 3 [4,19,102,103,104]. Zirconia ceramics are used as frameworks for all ceramic crowns and multi-unit prosthetics and also as monolithic full contour restorations [105] due to their physicochemical properties [106]. Yttria-stabilized tetragonal zirconia powders (3 mol%) (3Y-TZP) are regarded as the first generation of zirconia used for dental restorations, although 3Y-TZP has an opaque aspect [18,106]. The thickness of zirconia veneers is highly important for a good esthetic outcome, as thick zirconia negatively affects light transmission through the resin-matrix cement (Table 1). That also leads to low DC values of the resin-matrix cement and consequently to color instability and low microhardness values [19]. Based on the data of a previous study, a decrease in the light transmission occurs through 3Y-TZP at variable thickness, although the lowest decrease was recorded for a thickness of 0.2 mm and the highest one was for a thickness of 1.65 mm [60]. Another study evaluated the effect of thickness and curing time on the shear bond strength of a resin-matrix cement. Three different thicknesses of 1, 1.5, and 2 mm were analyzed. The polymerization time varied from 20 up to 40 s. The thickness of zirconia increased and the shear bond strength of the dual-cured cement decreased. An increase in the polymerization time to 40 s led to a reliable bond, thicker than 2 mm, between the ceramic and the dual-cured cement [107].

However, the brittle nature of ceramic restorations remains a problem [108]. Resin-matrix composites and PICN manufactured by CAD-CAM become alternative composites to ceramic restorations. Such materials combine the positive features of ceramic materials and resin-matrix composites, providing a hybrid material with adequate microhardness which become less aggressive against teeth. However, composite materials have lower fracture toughness when compared to glass ceramics [104]. The polymerization of resin-matrix cements through composite blocks manufactured by CAD-CAM depends on the restorative material thickness (1.5–2.7 mm), LCU type, and microstructure. Nevertheless, a dual-cured resin cement is required for thicker restorations, as high irradiance LCU has only a limited effect on the maximum thickness of resin-matrix composite blocks [7]. A previous study compared three different thickness (1, 2, and 3 mm) of three different materials, including leucite-based-glass-ceramic, zirconium-reinforced lithium silicate glass-ceramic, and PICN [109]. The findings were important for selecting the material and the thickness that are highly important for clinical success when dual-cured resin-matrix cements are used for cementation [109]. The use of light-cured resin cements is limited to specific clinical cases such as veneer cementation or thin indirect restorations, i.e., indirect restorations in which the thickness and restoration color do not interfere with the light-curing process [110]. Thus, light-cured resin cements should not be used if the restoration is thicker than 3 mm to prevent inadequate polymerization [36]. On the other hand, dual-cured resin matrix cements are indicated for use in thicker indirect restorations [16,105,111]. For lithium disilicate glass ceramics with thicknesses between 0.6 and 1.5 mm, the use of light-cured or dual-cured resin-matrix cements had no effect on the final DC for two tested resin-matrix cements (NX3^TM^, Kerr, and Choice 2^TM^, Bisco). Regarding the thickness of lithium disilicate-reinforced glass ceramics, the results showed that a thick layer did not significantly attenuate the light transmission from LCU [38].

The light transmittance of dual-cured resin cements is affected by different factors, including irradiation time, light irradiance, light transmission, and light-curing protocols, as well as type, thickness, and shade of the materials, as shown in Figure 3 [16,112,113,114]. Regarding the light curing time for indirect restorations with 2 mm thickness or above, a minimum of 40 s of light-curing is recommended [16,52,114]. A previous study concluded that using a high LED irradiance for shorter times of light activation resulted in the lowest DC and maximum polymerization rate of resin-matrix cements in ceramics [90]. The light-curing protocol is very important when luting ceramic veneers. The exposure time and the percentage of light transmitted through the material can affect the amount of energy delivered through the resin cement. Consequently, that influences the DC and the mechanical properties of the resin-matrix cements such as microhardness [52,115]. Regarding the light exposure time, a previous study reported differences between single- and multi-emission LCUs through the analysis of two resin-matrix cements with different photoinitiators (AllCem^TM^ and Variolink^TM^) [52]. The results revealed that the highest DC and microhardness were achieved in both resin-matrix cements when the samples were polymerized individually for 40 s [52]. In both resin-matrix cements with different photoinitiators, the DC values were not significantly affected by the LCU [52]. Another study analyzed the effect of single- and multiple-emission peak LCUs on the DC and microhardness of two shades (A2 and A4) of resin-matrix cement (Variolink II^TM^) under a 1 mm-thick Empress Esthetic ceramic A2. After light curing for 20 s, the resin-matrix cements were polymerized either with a single- or multiple-emission peak light irradiance [116]. The radiant exposure reaching the resin-matrix cement was determined by incident irradiance, exposure time, ceramic type, and ceramic thickness. The exposure time was the most consistent parameter affecting the mechanical properties of the resin-matrix cement. For light curing resin-matrix cements, an exposure time of 20 s is recommended [117]. Two resin-matrix cements (RelyX Ultimate^TM^ and Variolink^TM^) were assessed in resin-matrix composite restorations. Despite the dual-curing mechanism, the resin-matrix cements were either self-cured or light-cured. The self-cured components were not able to compensate the lower light irradiance and a decrease in the amount of resin-matrix composite overlay was not effective for monomer conversion. The light irradiance guaranteed the mechanical properties of resin-matrix cements [72].

A previous study measured the DC percentage and light irradiance of light-cured and dual-cured cements. Four types of indirect restorative materials with 1.5 mm thickness were assessed: Vita Enamic^TM^, Vita Suprinity^TM^, GC Cerasmart^TM^, and Degudent Prettau Anterior^TM^. The DC of dual-cured and light-cured resin-matrix cements (RelyX Ultimate Cliker^TM^) were analyzed after being light cured for 40 s with a LED LCU using two polymerization modes, soft-start and pulse delay, or using a QTH LCU for 20 s. The results showed a higher DC percentage of 62.9% for the dual-cured resin-matrix cement polymerized by using the LED soft-start mode exposed to direct light 1 day after polymerization. The dual-cured resin-matrix cement showed a DC of 63% with an LED LCU using the soft-start method on 1.5 mm restorations. The DC percentage of all ceramic restorations was affected by the amount, size, and type of crystal. Additionally, the microstructure and chemical content of the resin-matrix luting cement and the type of curing light were determinant factors for the DC [50,118]. Light scattering through ceramic restorations and light reflection caused a reduction in light transmittance to the resin-matrix cement below the restoration [90,119]. The use of light- and dual-cured resin cements is suitable for cementation of restorations up to 1.0 mm thickness, while the use of a dual-cured resin-matrix cement is recommended in restorations of over 1.0 mm thickness [120]. 

The light-curing parameters have limitations regarding the thickness and microstructure of different restorative structures, and therefore dual polymerization of resin-matrix cements is recommended. The light-curing parameters must be adjusted to the different restorative materials and equipment. Additionally, differences in light irradiance and wavelength from light curing units affect the polymerization of traditional and novel resin-matrix cements. The development of restorative materials, resin cements, and photoinitiator systems should be correlated with an emphasis on the polymerization mode and equipment used by clinicians. Details on the microstructure, chemical composition, and thickness of onlays, inlays, and prosthetic crowns are missing in several previous studies. A wealth of information on restorative materials and resin-matrix cements is crucial for the long-term success of dental restorations. Future studies should be performed regarding the chemical composition, thickness, and microstructure of indirect restorative interfaces cemented with resin-matrix cements.

## 4. Concluding Remarks

Within the limitations of the previous studies, the following conclusions can be drawn. The degrees of conversion of dual-cured resin-matrix cements were higher than those recorded for light-cured resin cements. The content, type, and size of inorganic fillers of the resin-matrix cements affected their polymerization. Absorption and scattering within the material are the main factors associated with light attenuation. The organic matrix components, such as the photoinitiator systems, chemical composition and viscosity of the methacrylate-based monomers, have a direct effect on the polymerization reaction. Considering indirect restorations, high translucency materials allow light transmission towards the resin-matrix cement, leading to a high degree of conversion of monomers. The degree of conversion of monomers decreases for restorative opaque materials with thickness higher than 1.5 mm. Light scattering through ceramic restorations causes a reduction in light transmittance to the resin cement below the restoration. A high light irradiance and exposure time must be selected for opaque materials such as traditional zirconia to achieve the required energy for the polymerization of the resin-matrix cement. The soft-start polymerization method showed the highest degree of conversion in dual-cured cements when compared to light-cured cements. All these variables must be considered in clinical practice, mainly when the clinicians select the type of resin-matrix cement, the indirect restoration, and the light curing unit. The effects of light transmittance on the polymerization of resin-matrix cements should be clarified in further studies concerning their mechanical performance and biocompatibility. 

## Data Availability

The datasets used and/or analysed during the current study available from the corresponding author on reasonable request.
